# Supplementary material for: Identifying the outbreak signal of COVID-19 before the response of the traditional disease monitoring system
Source: PLoS Negl Trop Dis. 2020 Oct 1;14(10):e0008758. doi: 10.1371/journal.pntd.0008758 (PMC7553315; doi:10.1371/journal.pntd.0008758)
Supplement: Attachment — Submitted filename: Reply.doc [file pntd.0008758.s001.doc]

Dear editor,

We are grateful for your critical comments and thoughtful suggestions. Based on these comments and suggestions, we have made extensive modifications to the original manuscript. We also asked the "AJE" commercial service to help edit our paper. All changes made to the text are highlighted or with track changes. We hope that the new manuscript will meet your journal’s standards. Below you will find the point-by-point responses to the reviewers’ comments/questions:

With this letter, we would like to confirm that the material has not and will not be offered elsewhere for possible publication, as long as it is under your consideration. We also confirm that all the contributing authors have reviewed this revision and concur with the revised submission made by the corresponding author. We are looking forward to hearing from you soon.

**Reviewer #1: 1. How can the confounding factors be controlled for "the Baidu search engine ([http://index.baidu.com/v2/#/)](http://index.baidu.com/v2/#/)) to analyze Baidu Index for the keywords of “pneumonia” and “SARS” being searched from 1 November, 2019, to 1 February, 2020. " ?**

Response:

Thanks for your comments. The web-based search for key terms or ILI surge counts in relation to the emergence of COVID-19 may be attributed to potential confounders. It can also be affected by media coverage, the population’s knowledge, or the degree of information disclosure. We have discussed it in the revised manuscript as the following:

“In our study, several potential limitations should not be neglected. First, the web-based search for key terms or ILI surge counts in relation to the emergence of COVID-19 may be attributed to potential confounders. Second, the observed ILI surge may represent more than just SARS-CoV-2-infected patients. Whether ILI surveillance data could be used for the signal of the EIDs without dominant features of COVID-19, such as cough and fever, is unclear. Third, the web-based search can be affected by media coverage, the population’s knowledge or the degree of information disclosure.”

**2. Please show the approved documents for ethical approval.**

Response:

Data of this study were extracted from a public database. No individual information was published in this paper. Therefore, this study is exempt from ethical approval. This manuscript has also been reviewed by the Academic Committee of the School of Public Health, Nanjing Medical University.

**Reviewer #2: 1. Please confirm that most of the important literature has been included in the paper.**

Response:

We have added more recently published literature in the revised version.

**2. The analysis is mainly descriptive, and the authors need to use statistical testing to come to the conclusion if possible.**

Response:

We have added statistical analysis on the ILI cases in the revised version.

**3. This study is an aggregation analysis, and only need an exemption approval of ethics.**

Response:

Data of this study were extracted from a public database. No individual information was published in this paper. Therefore, this study is exempt from ethical approval. This manuscript has also been reviewed by the Academic Committee of the School of Public Health, Nanjing Medical University.

**Reviewer #2: 1. The analysis is reasonable. Is there a possibility of analyzing the spatial correlation of ILI and COVID-19? The current correlation may not be enough to support the conclusions.**

Response:

Thanks for your suggestion. Due to the data limitation, we did not analyze the spatial correlation of ILI and COVID-19.

**2. The correlation of ILI and COVID-19 may be from a co-occurring event, that is, the two events may be occurring simultaneously with seasonality, meaning that the next peak of ILI might not be a good signal of COVID-19.**

Response:

Thanks for your comments. We hypothesized that the excessive ILI or abnormal increase of ILI could be an early signal of emerging infectious diseases, especially for airborne transmitted diseases, not specific to COVID-19.

**3. Is Figure 1A completed by the authors themselves? Otherwise, they need to declare the information and cite it.**

Response:

We've redrawn Figure 1 and cited the reference.

**Reviewer #1: How can these abnormal and rapid increase in the number of influenza cases be related to COVID-19, why not other infectious diseases?**

Response:

Thanks for your comments. We hypothesized that the excessive ILI or abnormal increase of ILI could be an early signal of emerging infectious diseases, especially for airborne transmitted diseases, not specific to COVID-19.

**Reviewer #2: Conclusion needs to be more specific and give detailed information of the measures we can take to detect the epidemics.**

Response:

Thanks for your comments. We have revised the part of the conclusion.

**Reviewer #2: This paper performed a comparative study to discuss the feasibility of early warning of the outbreak of COVID-19 in China based on the influenza surveillance data and internet Baidu search index to evaluate the timelines of the alert signals in comparison with the traditional case reporting system and official response, and suggested that monitoring abnormal spike of influenza-like cases or identifying online search peak of key terms could provide early signals of novel emerging infectious disease outbreak. Overall this paper provided very useful information about the early detection of COVID-19 epidemics. However, because of the lack of detailed analysis and specific conclusion, the authors need to put more further analysis in the manuscript in order to have a high impact on academic priority.**

Response:

We have added statistical analysis on the ILI cases in the revised version.

**Figure Files:**

**While revising your submission, please upload your figure files to the Preflight Analysis and Conversion Engine (PACE) digital diagnostic tool,** [**https://pacev2.apexcovantage.com**](https://pacev2.apexcovantage.com/)**. PACE helps ensure that figures meet PLOS requirements. To use PACE, you must first register as a user. Then, login and navigate to the UPLOAD tab, where you will find detailed instructions on how to use the tool. If you encounter any issues or have any questions when using PACE, please email us at figures@plos.org.**

Response:

We have uploaded figures to PACE to ensure that figures meet PLOS requirements.
